# Arthroscopic treatment of stable nonunion, unstable nonunion, or nonunion of the scaphoid with early degenerative radioscaphoid arthritis

**DOI:** 10.1186/s13018-023-03609-8

**Published:** 2023-02-21

**Authors:** Yin-chuan Shih, Chang-Chin Wu, Jui-Tien Shih

**Affiliations:** 1 Department of Orthopaedic Surgery, En Chu Kong Hospital, New Taipei City, Taiwan, ROC; 2 Department of Orthopaedic Surgery, National Taiwan University Hospital, Taipei, Taiwan, ROC; 3 Department of Orthopaedic Surgery, School of Medicine, National Taiwan University, Taipei, Taiwan, ROC; 4 Department of Biomedical Engineering, Yuanpei University of Medical Technology, Hsinchu, Taiwan, ROC; 5 Department of Orthopaedic Surgery, Armed Forces Taoyuan General Hospital, 168, Joing-Hsing R, LongTan County, Taoyuan, Taiwan, ROC

**Keywords:** Scaphoid nonunion, Wrist arthroscopy, Scaphoid nonunion advanced collapse (SNAC)

## Abstract

**Background:**

This study was designed to analyze the clinical follow-up results (minimum of 2 years) in patients with stable nonunion, unstable nonunion, or nonunion of the scaphoid with early degenerative radioscaphoid arthritis (Lichtman classification stage I–III) treated with arthroscopic osteosynthesis with autogenous bone graft.

**Methods:**

We retrospectively recruited 44 consecutive patients with scaphoid fracture nonunion treated with arthroscopy-assisted percutaneous internal fixation with autogenous bone grafts from January 2010 to November 2019. We recorded union and return to activity and analyzed data with regular clinical follow-up at a mean duration of 33 months (range 24–46 months). Clinical (i.e., visual analog scale pain score, grip strength, and range of motion), radiographic, and functional (Mayo Modified Wrist Score (MMWS)) outcomes at the final follow-up were compared with the preoperative assessments and analyzed in patients with different stages.

**Results:**

We confirmed union in 39 of the 44 patients (88.6%) after a mean 15.4 weeks post-operatively according to clinical examinations and standard radiography. All clinical parameters improved significantly. For the MMWS, there were 25 excellent and 14 good results. Of the 44 patients, 40 (90.9%) returned to work or sports activities at their preinjury levels. Comparisons of the outcomes between patients in different stages of scaphoid nonunion revealed no significant difference in the aspect of union rate, VAS pain score, and functional score improvement.

**Conclusions:**

Arthroscopic osteosynthesis with autogenous bone grafts is a reliable and minimally invasive method for achieving nonunion healing and improving clinical outcomes in stage I–III scaphoid nonunion.

*Level of Evidence*: Level IV, case series.

## Background

Scaphoid fractures are the most common carpal bone fracture, but the failure rates of their treatments have been reported to be 25–45% due to the unique vascularity, structure, and kinematics of these fractures [1, 2]. When scaphoid nonunion develops, the consensus of treatment is adequate debridement of nonunion bone gap and scar tissue to expose healthy, well-vascularized cancellous bone; appropriate bone grafting; and achievement of good stability of the fracture site with internal fixation [3–6]. Approach with fewer soft tissue injuries is preferred to preserve tenuous blood supply and key ligaments, which is concerned to cause scaphoid nonunion and instability [7, 8].

There have been a few studies reporting that arthroscopic treatment for scaphoid nonunion results in satisfactory outcomes [9–12]. However, most studies were small case series and focused on the early stages of scaphoid nonunion with minimal sclerosis without too much deformity. Here, we report the outcomes and technique details of scaphoid nonunion treated with an arthroscopic technique and extend the indication from stable scaphoid nonunion (Lichtman stage I) to unstable scaphoid nonunion (Lichtman stage II) and scaphoid nonunion with early degenerative radioscaphoid arthritis (Lichtman stage III).

This study was designed to analyze the clinical follow-up results for a minimum 2 years in patients with scaphoid nonunion (Lichtman classification stages I–III) treated with arthroscopic osteosynthesis with autogenous bone graft. We hypothesized that arthroscopic osteosynthesis with autogenous bone grafts is a reliable and minimally invasive method for achieving bony healing and improving clinical outcomes in patients with stage I–III scaphoid nonunion.

## Methods

**Fig. 1:**
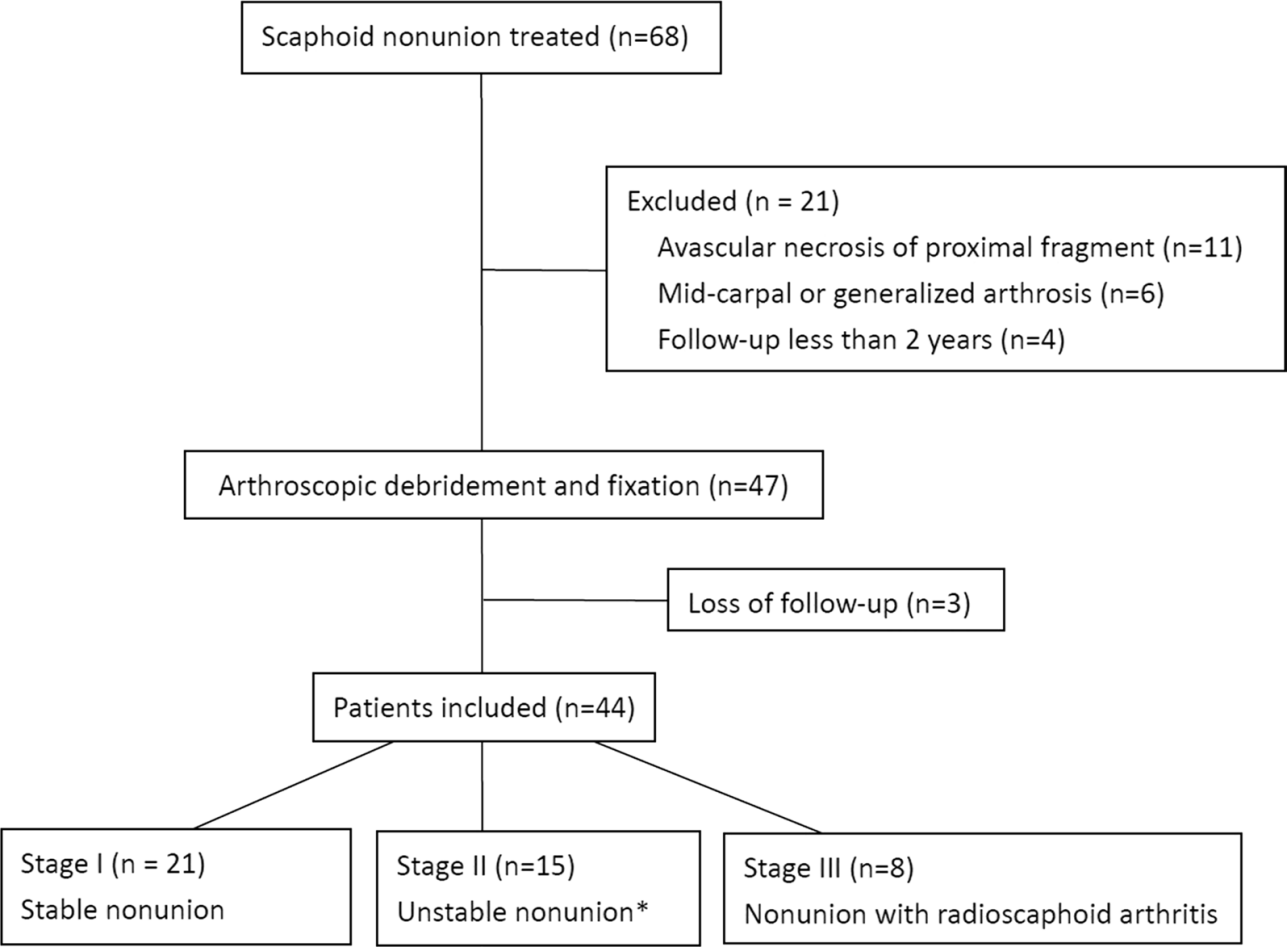
A Consolidated Standards of Reporting Trials flow diagram shows enrollment and analysis of this study. *Unstable nonunion defined as (1) Sclerosis, cystic change, or gapping and/or translation of more than 2 mm. (2) Lateral intrascaphoid angle (ISA) of more than 60°. (3) Radiolunate angle (RLA) of more than 10°; and. (4) Scapholunate angle (SLA) of more than 60° in preoperative radiographs

Approval for this retrospective study was obtained from our hospital’s institutional review board. From January 2006 to January 2017, a total of 47 surgeries performed on patients with scaphoid nonunion using arthroscopic-assisted osteosynthesis with autogenous bone graft and percutaneous internal fixation. Of the 47 patients, 44 were eligible to be included in this retrospective study for follow-up examination and radiographic review. Three patients were lost to follow-up because of military training. All patients had stage I–III scaphoid nonunion according to the Lichtman classification [13–16]. In stage I, there are stable nonunion without displacement, collapse, dorsal intercalated segment instability (DISI) deformity, or degenerative changes. In stage II, there are unstable nonunion with significant displacement (gapping or/and translation of more than 2 mm or volar collapse and DISI) [4, 17, 18]. In stage III, there are nonunion with early degenerative changes limited to the radioscaphoid joint with narrowing and pointing of the radial styloid. Patients with scaphoid nonunion with avascular necrosis, midcarpal, or generalized arthrosis including the radiolunate joint (stages IV and V), and less than 2 years of follow-up were excluded from this study (Fig. 1).

### Clinical evaluation

Clinical outcome evaluation was based on visual analog scale (VAS) pain score, range of motion, grip strength, return to work, and satisfaction with the operation during the follow-up. Modified Mayo Wrist Score (MMWS) questionnaire was completed by each patient during the preoperative and final follow-up. This system is commonly used to assess hand and wrist conditions and comprised 100 points divided into excellent (90–100 points), good (80–89 points), fair (65–79 points), and poor (less than 65 points) [19].

### Radiographic data

Standard preoperative radiographs were obtained in the anteroposterior, lateral, and oblique views, in addition to clenched-fist and radioulnar-deviation views of the affected wrist. Scaphoid nonunion was assessed radiographically by two hand surgeons (one had patients in the study, and one was an independent observer) and a radiologist. The stage of nonunion was classified using the Lichtman classification. All patients in our series underwent magnetic resonance imaging to evaluate the vascularity of the proximal fragment. If avascular necrosis of the proximal part was observed, they were excluded from this study. Postoperative radiographs were evaluated for the union and progression of arthritis in all patients, and changes in the scapholunate angle, radiolunate angle, and lateral intrascaphoid angle of the patients with Lichtman stages II and III were assessed as well. The consolidation of union was according to scoring system with callus formation and visibility of fracture line at four cortices on AP and lateral views [20]. Nine patients underwent computed tomography (CT) of the scaphoid in the sagittal and coronal projections to confirm union due to inconsistency of clinical symptoms and radiographic data; the scaphoid was evaluated to be united if the trabeculation across the site of the fracture could be seen on at least three long-axis sagittal CT images.

### Surgical procedure

#### Step 1: diagnostic arthroscopy

Under general anesthesia or axillary block, wrist arthroscopy was performed using a 1.9-mm-diameter arthroscope, two finger traps, and a traction device with 10–15-lb longitudinal traction. The radiocarpal and midcarpal joints were routinely explored through the 3–4, 6R, midcarpal radial, and midcarpal ulnar portals. Diagnostic arthroscopy was performed first with specific attention paid to the dorsal portion of the scapholunate interosseous ligament and the presence of full-thickness defects on the proximal pole of the scaphoid.

#### Step 2: fracture debridement and bone grafting

**Fig. 2:**
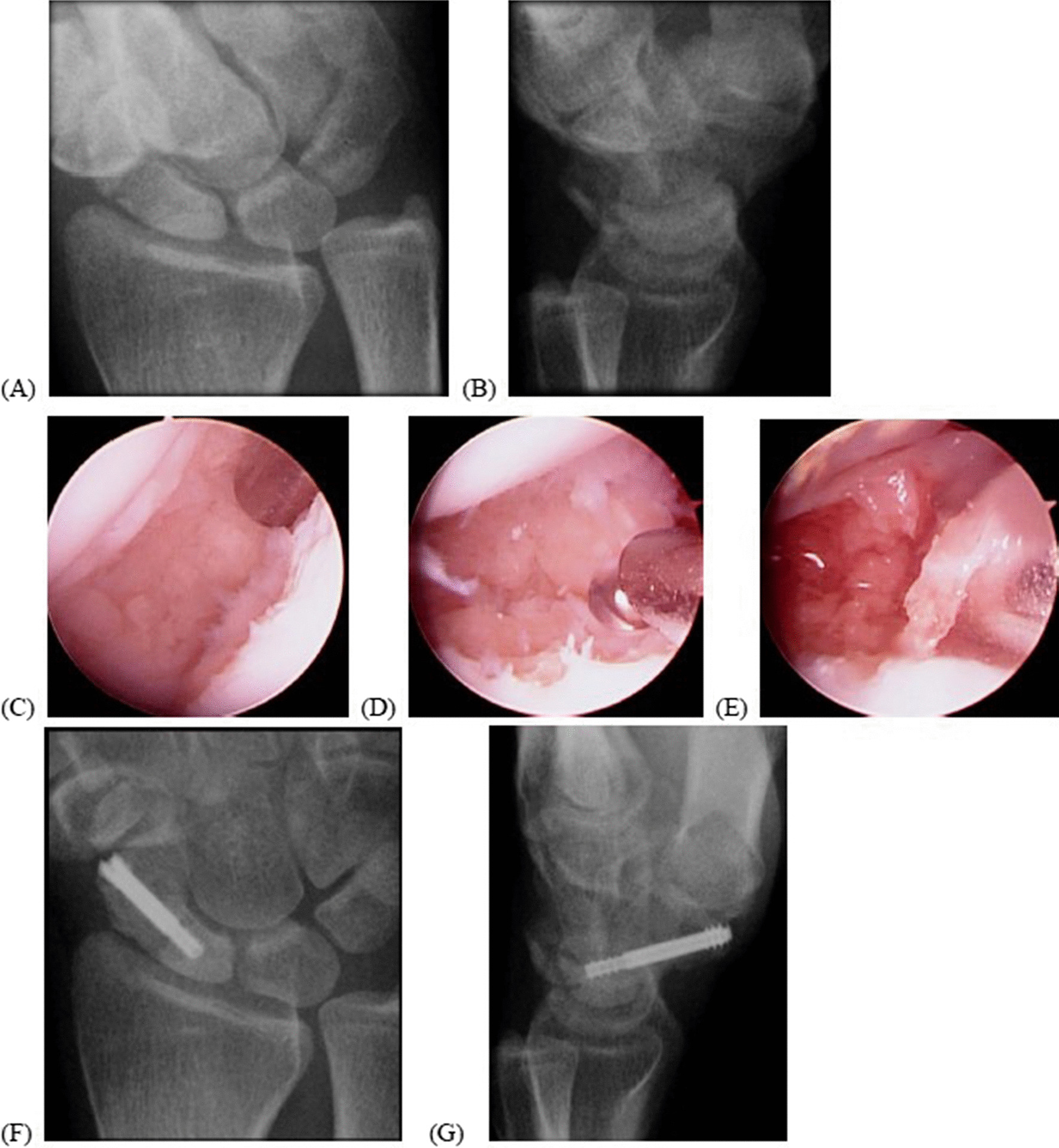
This is the case of a 21-year-old woman with right scaphoid nonunion, stage I, for 8 months (**A**, **B**). The patient underwent arthroscopically assisted percutaneous internal fixation with autogenous bone grafts (**C**, **D**, **E**). The scaphoid union was achieved at the 3-month follow-up (**F**, **G**)

**Fig. 3:**
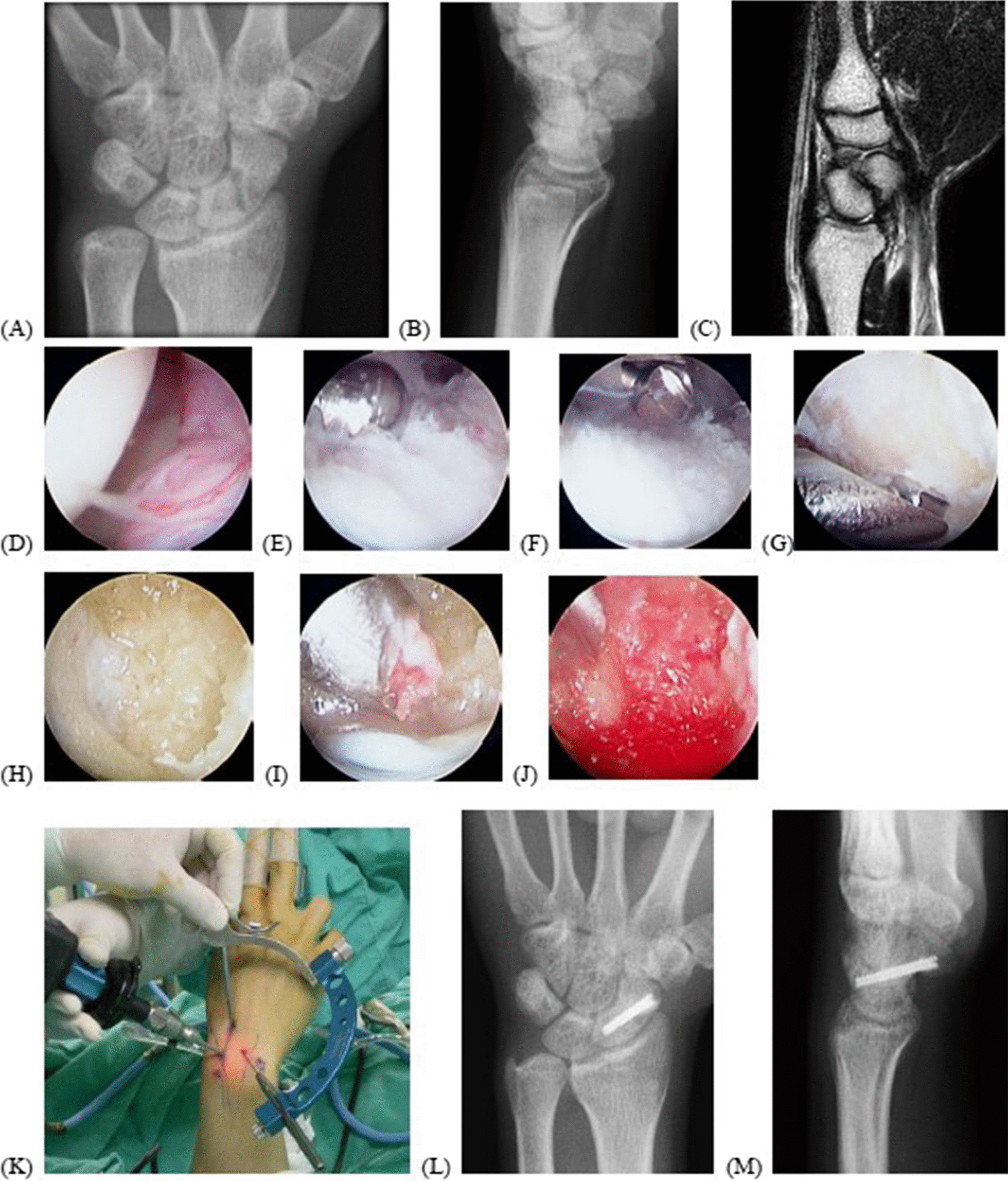
A 23-year-old male patient experienced trauma for 8 months with scaphoid fracture nonunion, stage II (translation/gapping of more than 2 mm), without proximal fragment avascular necrosis (**A**, **B**, **C**). Intraoperative arthroscopic photos from the midcarpal ulnar portal demonstrated fibrous nonunion (**D**), which was debrided and shaved (**E**, **F**, **G**). The fracture gap was filled with autogenous bone graft through the sheath of the burr (**H**–**I**), and there was the bleeder from the fracture fragment after off-pump of the water and tourniquet (**J**). The K-wire joystick through Micro Vector Drill Guide System could be used as joystick to help in the reduction (**K**). The scapholunate and radiolunate angles were corrected, and bony union was achieved after 3 months (**L**, **M**)

Midcarpal ulnar portal was used to identify the scaphoid fracture nonunion gap, and midcarpal radial portal was then created as working portal. For scaphoid waist and proximal nonunion, standard midcarpal radial portal allowed good working exposure; for distal third scaphoid nonunion, the working portal is modified toward scaphotrapeziotrapezoidal (STT) joint to reach nonunion site more easily. The fibrotic tissue of fracture surface was debrided using a power shaver and burr to expose the subchondral bone. The bleeder from the distal fragment of the scaphoid, a good sign of bony union, was checked with deflated tourniquet. Under dry arthroscopy, autogenous cancellous bone grafts taken from the distal radius or ilium crest depending on the gap of the nonunion were put densely into the fracture site through a 2.9 mm burr sheath (Figs. 2D, E and 3I, J).

#### Step 3: reduce the malalignment and DISI deformity

The reduction of the fracture fragment was attempted internally with k-wires introduced percutaneously with the Micro Vector Drill Guide System (Smith & Nephew, Andover, MA) into the proximal and distal fracture fragments as joysticks (Fig. 3K). In patients with type II nonunion with DISI instability, the hyper-extended lunate and proximal fragment of the scaphoid were reduced to the neutral position with wrist flexion, and the radiolunate joint was temporally fixed using a K-wire (Linscheid maneuver) [12]. After scaphoid deformity was corrected and fixed, the k-wire of radiolunate joint was removed. The positioning and alignment of the K-wires were examined fluoroscopically.

#### Step 4: soft tissue and interosseous ligament debridement and thermal shrinkage

For soft tissue injuries, if there was any interosseous ligament tear or triangular fibrocartilage complex (TFCC) tear, the ligament was debrided using a power shaver or thermal probe.

#### Step 5: arthroscopic radial styloidectomy for stage III scaphoid nonunion

Patients with stage III nonunion underwent arthroscopic radial styloidectomy. A synovial power resector through 1–2 portal was used to perform a radial-sided synovectomy, which permitted the visualization of the entire radius. The origin of the radioscaphocapitate ligament was visualized to avoid damage.

#### Step 6: apply internal fixation

After that, we released traction and applied headless compression screws or K-wires through a volar percutaneous incision to fix the fracture site under fluoroscopy according to the stability of the scaphoid fracture. The screw length and stability of fracture gap were confirmed by arthroscopic exploration and visualization The patients in Lichtman stage III underwent non-parallel K-wire fixation initially to avoid over-compression of the unstable fracture, and after 4–6 weeks, they would undergo the removal of the K-wires, which would be changed to a headless compression screw for fixation with fluoroscopic guidance.

#### Step 7: postoperative care

All patients underwent screw or K-wire fixation had thumb-spica casts immobilization for 6–8 weeks. Active joint exercises were begun on cast removal, and unrestricted activities resumed approximately 6 weeks after cast removal. All patients were monitored regularly at the outpatient clinic.

### Statistical analysis

The normality of the data was verified using the Kolmogorov–Smirnov test, which showed that the data were nonparametric. The Wilcoxon signed-rank test was used to compare preoperative and postoperative values. Outcomes analysis between different group was analyzed by Fisher’s exact test, Kruskal–Wallis test and post hoc tests [21]. Statistical correlation was analyzed deeming *p*-values of less than 0.05 to be statistically significant. The minimal clinically important difference threshold of 13.5 points for the Disabilities of Arm, Shoulder, and Hand score of each patient was also evaluated and considered important by the patients [22, 23].

## Results

The 44 patients included in this study were followed for a mean duration of 33 months (range 24–46 months). The mean time from injury to surgery was 8.5 months (range 4 months to 2.1 year). Nine injuries were in the nondominant hands, and 21 injuries were work-related. Using the Lichtman classification, the nonunion were categorized as stage I in 21 patients, stage II in 15 patients, and stage III in eight patients. Moreover, 40 patients had scaphoid waist nonunion, and the remaining four patients had proximal pole nonunion. Among stage III patients, eight had full-thickness articular cartilage loss over the radial styloid dorsally and that all patients had a normal midcarpal joint. According to the arthroscopic findings, 14 patients had laxity of the scapholunate ligament, seven patients had partial lunotriquetral tears, and 15 patients had triangular fibrocartilage complex superficial tears (Table 1).

**Table 1:** Baseline Characteristics of Patients Subsets

Variables	Total number = 44
Age range, years	20 to 46
Dominant/nondominant, *n*	35/9
Duration of Symptoms, month	8.5 (range 4–25)
Follow-up, month	31 (range 24–46)
Work-related injury Gap space of fracture nonunion, mm	21 1.8 (range 0.7–2.3)
*Nonunion site, n*	
Proximal	4
Waist	40
Distal	0
*Lichtman classification, n*	
Stage I	21
Stage II	15
Stage III	8
*Associated injury*	
TFCC injury	15 (34.1%)
Scapho-lunate injury	14 (31.8%)
Lunotriquetral injury	7 (15.9%)
*Autogenous bone graft*	
Distal radius	29
Iliac bone	15
*Fixation method*	
Headless compressive screws	36
K-wire	8

In our series, by carefully applying arthroscopic osteosynthesis with bone graft and assisted reduction and internal fixation with headless compression screw (types I and II) or K-wires (type III), 39 patients (88.6%) achieved successful bony union in a mean duration of 15.4 weeks (range 10–24 weeks). At the last follow-up, the VAS pain score, grip strength, and functional scores significantly improved. According to the MMWS, 25 patients had excellent wrist function and 14 patients had good wrist function. Forty patients returned to work or sports at the preinjury level. However, postoperatively, five patients still had nonunion and poor results without improvement in grip strength. That is, three patients had type II nonunion and two patients had type III nonunion. No arthritis progression and operation-related complications, including infection and implant loosening or breakage, were observed at the last follow-up. The radiographic alignment, including the scapholunate, radiolunate, and lateral intra-scaphoid angles, in stage II and stage III patients significantly improved (Table 2). Comparisons of the outcomes between patients in different stages of scaphoid nonunion revealed no significant difference in the aspect of union rate, VAS pain score, and functional score improvement (Table 3).

**Table 2:** Clinical and radiographic outcomes

Variables	Preoperative	Last follow-up	*p*-value
Union rate, total, *n* (%)	–	39 (88.6%)	–
Returned to work or sports activities at the preinjury level	–	40 (90.9%)	–
Interval of Achieved Bony Union (weeks)	–	15.4 (10–24)	–
VAS pain score	5.3 (1.2)	1.2 (0.9)	< .0001
Grip strength % of healthy side	56.8%	88.7%	< .0001
MMWS (points) Excellent Good Fair Poor	50.2 (9.5) 0 0 0 44	88.5 (10.8) 25 14 0 5	< .0001
DASH score (points)	32.3 (5.7)	5.9 (4.2)	< 0.001
Flexion–extension arc, °	103.5 (15.9)	106.4 (13.7)	0.242
SLA in stage II and III, ° (*n* = 23)	54 (10.8)	47.5 (8.1)	< .0001
RLA in stage II and III, ° (*n* = 23)	6.2 (3.2)	4.3 (2.7)	< .0001
LISA in stage II and III, ° (*n* = 23)	34 (14.5)	27.8 (9)	0.004

Continuous values expressed as mean (standard deviation)

*MMWS* Mayo modified wrist score; *DASH* Disabilities of Arm, Shoulder and Hand

*SLA* scapholunate angle; *RLA* radiolunate angle; *LISA* lateral intra-scaphoid angle

**Table 3:** Outcome Analysis Between Groups with Different Stages of Scaphoid Nonunion

	Stage I (*n* = 21)	Stage II (*n* = 15)	Stage III (*n* = 8)	*p*-value
Union, *n* (%)	21 (100%)	12 (80%)	6 (75%)	0.071
Returned to work or sports activities at the preinjury level, *n* (%)	21 (100%)	12 (80%)	7 (87.5%)	0.112
VAS pain score, mean change (95% CI)	4.2 (3.96–4.44)	3.9 (3.48–4.32)	4.4 (3.54–5.24)	0.245
MMWS (points), mean change (95% CI)	40.1 (35.1–45.1)	37.8 (32.8–42.8)	36.5 (25.4–47.5)	0.676
DASH score, mean change (95% CI)	27.4 (25.56–29.24)	27.9 (25.2–30.7)	26.0 (23.90–28.09)	0.561

*CI* confidential incidence

## Discussion

Scaphoid nonunion is challenging for even the most experienced wrist surgeon. There are a variety of treatment; however, the critical requirements of successful treatment include blood supply maintenance, nonunion debridement, fracture reduction, appropriate bone grafting, and rigid internal stabilization [4, 24]. Recently, minimally invasive techniques can be used to satisfy the aforementioned critical requirements for healing scaphoid nonunion as long as those requirements are not compromised [9–12]. It is not clear whether the formal open exposure wound affect the treatment outcome of scaphoid nonunion, but a minimally invasive approach with wrist arthroscopy assistance could theoretically minimize postoperative stiffness and maximize functional outcomes. The open exposure allows traditional cortical iliac crest wedge grafts or vascularized bone grafts, but inevitably disrupt wrist capsule and key ligaments. Although a recent retrospective comparative study has not shown any significant differences in clinical and radiological outcomes at a minimum follow-up duration of 2 years [25], it seems intuitive that the dissection required for placing these bone grafts would lead to greater postoperative morbidity than small percutaneous stab incisions.

In this study, arthroscopic treatment of scaphoid nonunion showed a satisfactory union rate and clinical outcomes and showed several advantages, including minimal soft tissue injuries, easy access to the nonunion site, and easy concomitant intra-articular evaluation. In the Lichtman classification, scaphoid nonunions were classified into five stages [13–16]. Avascular necrosis, midcarpal arthritis (stage IV), and generalized arthritis with radiolunate joint involvement (stage V) are contraindication of arthroscopic treatment. We explored the indication of arthroscopic management to Lichtman stage I–III scaphoid nonunion with different treatment strategies.

In stage I, stable scaphoid nonunion without displacement or arthritis could be treated with careful debridement of the nonunion site to subchondral bone exposure or best with bleeding. Non-vascularized bone graft from the iliac crest or distal radius could be easily inserted into the fracture site using a burr sheath. Stable fixation was achieved using percutaneous compression screws (Fig. 2). All patients (100%) in this stage had bony union and improved clinical outcomes. Most studies have agreed that arthroscopic management of scaphoid nonunion without severe gapping/deformities or arthritis was effective and as good as or better than those reported in a series of open approach non-vascularized bone grafting (80–95% union rate) [26–28].

In stage II, scaphoid nonunion was unstable or displaced with a substantial bone gap. The K-wire joystick technique or Linscheid maneuver could achieve good reduction [12]. In our series, stage II patients have satisfactory clinical outcomes and 80% union rate. The radiographic parameters all significantly improved. The comparative study showed that the open approach group had better radiographic alignment correction than the arthroscopic approach group, but with similar union rates and functional results [25]. However, Hsiung et al. have demonstrated that arthroscopic realignment and osteosynthesis of unstable scaphoid nonunion achieved good clinical and radiographic results [29]. The connection between alignment and clinical results remains unclear; however, Kim et al. have demonstrated that arthroscopic reduction and osteosynthesis of chronic unstable scaphoid nonunion were limited for restoring normal carpal alignment but had positive effects on the recovery of wrist function [17]. Substantial bone resorption could be filled with autogenous bone graft from the distal radius or iliac bone. Recently, Waitayawinyu T. et al. have reported satisfactory outcomes using arthroscopic treatment with olecranon bone graft [30]. In our series, satisfactory clinical results could be achieved by meeting the conditions of stable fixation, correction of scaphoid deformity and DISI, and sufficient bone graft without donor site complications (Fig. 2).

**Fig. 4:**
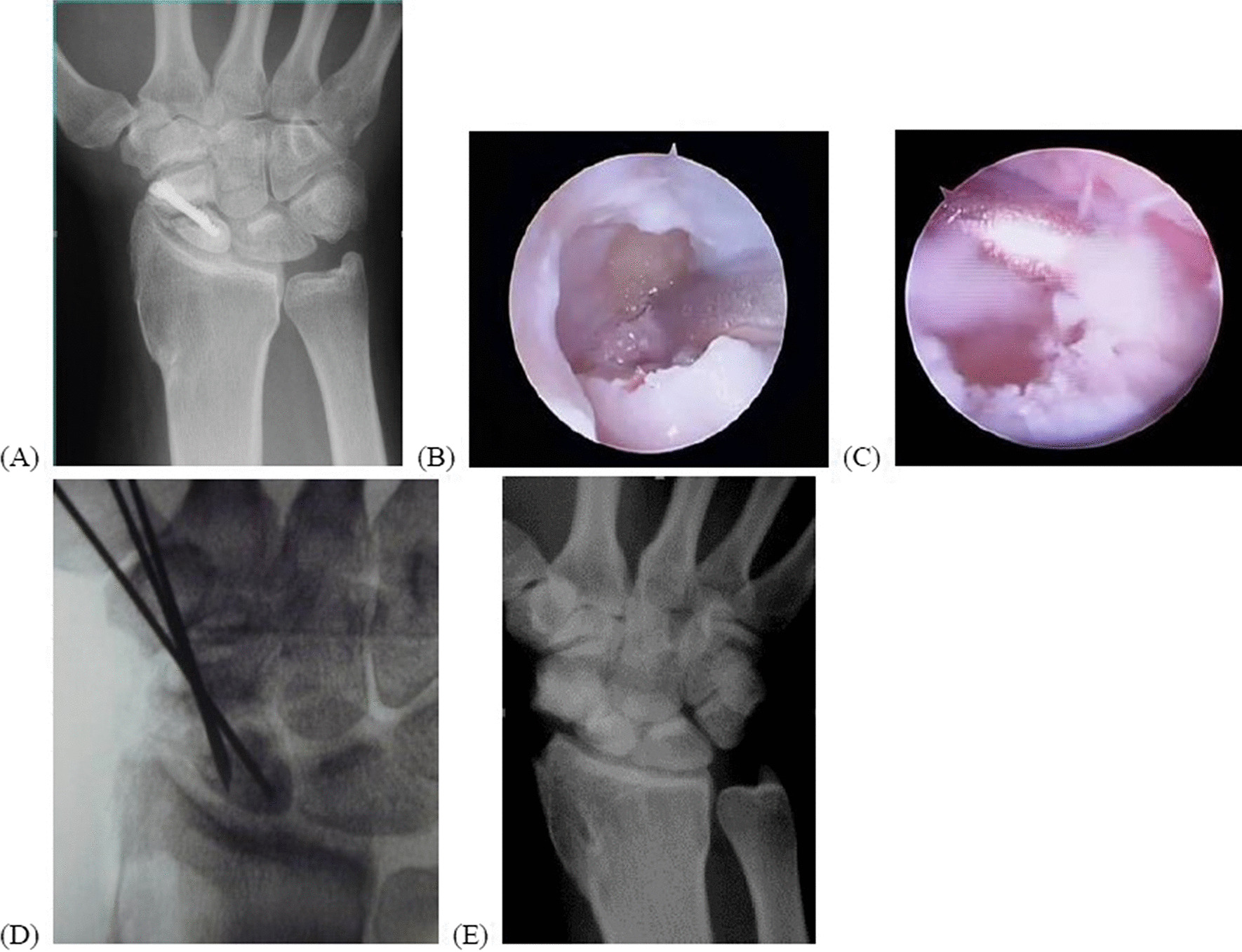
This 21-year-old male solider had right scaphoid nonunion, stage III, with radioscaphoid arthritis after the initial operation at another hospital 8 months ago (**A**). In addition to arthroscopic debridement and bone grafting (**B**, **C**), arthroscopic radial styloidectomy was performed to reduce radioscaphoid impingement. Multiple non-parallel K-wire fixation was performed to avoid over-compression (**D**). Bony union was achieved 8 months later (**E**)

Scaphoid nonunion may progress to a predictable pattern of arthritis as “scaphoid nonunion advanced collapse” (SNAC) [13]. In our series, eight patients had radioscaphoid arthritis due to deterioration instability and impingement between the radial styloid and distal fragment of the scaphoid (Lichtman stage III). They underwent arthroscopic radial styloidectomy, which was useful in reducing radial wrist pain caused by SNAC [31]. However, radial styloidectomy of more than 3–4 mm might cause iatrogenic ligament injuries and carpal instability [32, 33]. Recently, a cadaver study has shown that resection of more than 6 mm was associated with damage to radiocarpal ligaments [34]. Regardless, the arthroscopic technique allowed the identification of radiocarpal ligaments and the evaluation of the extent of arthritis during the procedure, and in our series, no patient had radiocarpal instability. For unstable reduction, K-wires were used for fixation, instead of compressive screws, to avoid over-compression (Fig. 4).

Of the 44 patients in this series, 39 achieved successful bony union in a mean duration of 15.4 weeks. Twenty-five patients had excellent wrist function, and 14 had good wrist function. Moreover, no complications were observed in our series. Five patients had poor results without improvement in grip strength because of persistent nonunion after surgery. Among them, four received vascularized bone graft with 1,2 intercompartmental supraretinacular artery pedicle through the dorsal approach to correct the deficiency, and these cases achieved bone union after 6–8 months. Two of the five patients (one in stage II and one in stage III) are smokers with a smoking history of more than 5 years, which may have reduced the bone-healing capacity [35, 36].

Arthroscopically treatment of scaphoid nonunion is a technically and instrumentally demanding procedure but a reliable treatment option for patients with early-staged (stage I to III) scaphoid nonunion without avascular necrosis of proximal fragment. This procedure has additional advantages of concomitant soft tissue evaluation, direct visualization of the nonunion surface and stability of the synthesis, and minimal morbidity. Most patients had good subjective results and significantly improved grip strength with the described method of arthroscopically assisted reduction and bone grafting techniques.

### Limitations

This study had some limitations. First, the number of patients with stage III nonunion was small and three patients were lost to follow-up due to military training, and these may lead to performance bias. Second, we did not have a control group of patients treated with an open procedure to compare the clinical and radiographic results. Moreover, scaphoid fracture nonunion is a complex challenging, so heterogeneous treatments including various methods for fixation and different bone grafting techniques are unavoidable. Regardless, the patients could perform good functional and high satisfaction rate at the follow-up.

## Conclusions

Arthroscopic osteosynthesis with autogenous bone grafts is a reliable and minimally invasive method for achieving bony healing and improving clinical outcomes in patients with stage I–III scaphoid nonunion.

## Data Availability

All the datasets are presented in the main manuscript and Tables 1, 2 and 3.
